# Words and Deeds

**Published:** 1921-01-15

**Authors:** 


### WORDS AND DEEDS.

Truth, said the American Bryant, will survive if run over by a locomotive, while error dies of lockjaw if it pricks its little finger. Nowadays we are fast getting proofs of the correctness of this proposition. During the war truth was burked on patriotic grounds. And the lowering of moral tone which, contrary to the expectation of superficial thinkers, followed the war, has encouraged more than one section of the Press to continue its policy of—to be plain—seeking untruths and ensuing them. Now, the influence of the Press upon public opinion is so strong that some medical men even, who mix much with the public, have slipped into a highly inconsistent way of talking and thinking; even certain leaders of the profession show remarkable cases of this unfortunate infection. Like the pressman, during the war and after the war they swore roundly that English, not Continental, medicine leads the world. Yet, as the French say, one cannot pay oneself with words for ever; the time comes for action, and action has to be based upon truth—that truth, that universal good, which Aristotle optimistically said every man desired to know, whether or not he desired to act upon it.

Our readers may be surprised at the ruthlessly candid tone of the above, so accustomed has the eye become to the smooth message that all is well. We are not, however, the first medical journal to deem that the time has come to speak out. In a recent leading article in the *Lancet* there are some very plain words, all the plainer as coming from a source never suspect of undue sympathy with what may be generally called the opinion of the Left. "Could we but flatter ourselves," says the writer, "that our backwardness was confined to a highly specialised branch of therapeutics, of value only to a few patients, we might yet enjoy the happiness of self-complacency. But at the risk of further wounding our national pride we would quote the opening lines of a recent review in a Danish journal." This review mentions a new proof, "added to many others," of the isolation and encapsulation of the British island kingdom from the rest of the world, not least in matters of science. A medical controversy waged on the Continent, and settled, twenty years ago, seems only now to have reached England, where, undisturbed by earlier investigations on the subject, the question is now being discussed. The question referred to is a urological one, and the highly specialised branch of therapeutics mentioned in the article is in connection with tuberculosis. But the phenomenon may be seen throughout the whole of medicine. Look at the discussion on bronchoscopy in the *Archiv für Laryngologie*, to be followed at a five-year interval by a working over of the exhausted ground in British journals.

Our contemporary well urges that a better knowledge of modern languages is required amongst specialists at home, so that the equipment of British doctors in this respect should be brought up to that of their colleagues on the Continent. Which of our hospitals, it asks, insists on applicants for honorary appointments possessing a knowledge of more than one modern language? We agree that a new regulation to this effect might in a few years effect marvels, especially in the provinces: and not the least benefit would be this, that medical literature would be saved the addition of those countless articles that, unknown to the well-meaning authors, contain nothing new at all. The *Lancet* writer does not end on a conciliatory note, he does not try to tone down his beginning, for his last word is that the solution of this problem is imperative "lest, in certain aspects of medicine, we become the China of the West." The beginning of wisdom here, we venture to think, is a recognition, and a wide recognition, of the facts thus suddenly and forcibly disclosed.

